# Monte Carlo Study of Radiation Dose Enhancement by Gadolinium in Megavoltage and High Dose Rate Radiotherapy

**DOI:** 10.1371/journal.pone.0109389

**Published:** 2014-10-02

**Authors:** Daniel G. Zhang, Vladimir Feygelman, Eduardo G. Moros, Kujtim Latifi, Geoffrey G. Zhang

**Affiliations:** 1 University of California, Berkeley, California, United States of America; 2 Radiation Oncology, Moffitt Cancer Center, Tampa, Florida, United States of America; University Paul Sabatier, France

## Abstract

MRI is often used in tumor localization for radiotherapy treatment planning, with gadolinium (Gd)-containing materials often introduced as a contrast agent. Motexafin gadolinium is a novel radiosensitizer currently being studied in clinical trials. The nanoparticle technologies can target tumors with high concentration of high-Z materials. This Monte Carlo study is the first detailed quantitative investigation of high-Z material Gd-induced dose enhancement in megavoltage external beam photon therapy. BEAMnrc, a radiotherapy Monte Carlo simulation package, was used to calculate dose enhancement as a function of Gd concentration. Published phase space files for the TrueBeam flattening filter free (FFF) and conventional flattened 6MV photon beams were used. High dose rate (HDR) brachytherapy with Ir-192 source was also investigated as a reference. The energy spectra difference caused a dose enhancement difference between the two beams. Since the Ir-192 photons have lower energy yet, the photoelectric effect in the presence of Gd leads to even higher dose enhancement in HDR. At depth of 1.8 cm, the percent mean dose enhancement for the FFF beam was 0.38±0.12, 1.39±0.21, 2.51±0.34, 3.59±0.26, and 4.59±0.34 for Gd concentrations of 1, 5, 10, 15, and 20 mg/mL, respectively. The corresponding values for the flattened beam were 0.09±0.14, 0.50±0.28, 1.19±0.29, 1.68±0.39, and 2.34±0.24. For Ir-192 with direct contact, the enhanced were 0.50±0.14, 2.79±0.17, 5.49±0.12, 8.19±0.14, and 10.80±0.13. Gd-containing materials used in MRI as contrast agents can also potentially serve as radiosensitizers in radiotherapy. This study demonstrates that Gd can be used to enhance radiation dose in target volumes not only in HDR brachytherapy, but also in 6 MV FFF external beam radiotherapy, but higher than the currently used clinical concentration (>5 mg/mL) would be needed.

## Introduction

Magnetic resonance imaging (MRI) is often used in tumor localization for radiotherapy treatment planning, and gadolinium-containing materials are often applied to enhance contrast for tumor volumes. Recently, polymeric micelles were developed to target cancers [1], [2]. The concentration of gadolinium used for MRI is reported to be about 2.4 mM [3].

In addition to imaging, using gadolinium-containing materials as radiosensitizers have been reported. Motexafin gadolinium (MGd) is a chemotherapeutic drug that selectively targets tumor cells and mediates redox reactions, generating reactive oxygen species [4], [5]. Also, as an avid electron acceptor, MGd depletes the pool of DNA repair substrates that are therefore unavailable to repair the oxidative damage to DNA induced by radiation [6], [7]. It also increases intracellular oxygen levels, thereby potentially overcoming hypoxia and allowing “fixation” of radiation damage [8]. In clinical trials, the plasma concentration was up to 77.1 µg/mL with a single intravenous administration of 6.3 mg/kg MGd [9].

In radiotherapy, radiation dose is delivered to the target volume to kill tumor cells. In treatment planning, radiation beams are arranged to aim the dose to the target volume and spare the surrounding normal tissues as much as possible. With higher dose in the target volume, thus better therapeutic ratio, dose escalation can be higher, which would generate better clinical outcome. Therefore, dose enhancement in the target volume has been a hot topic recently in radiotherapy. There have been several radiation dose enhancement studies in which tumor-targeted nanoparticles are used [10]. The concentration of gold-nanoparticles in the targeted tumor volume can reach 7 mg/mL [11]. Gadolinium has been reported to enhance dosage in brain tumor in microbeam radiation therapy [12]. In the microbeam study, beam energy ranged from 65 to 200 keV. Another Monte Carlo study using MCNPX code also showed that gadolinium enhances dose absorption in brachytherapy by up to 106% at 30 mg/mL concentration, although not as much as gold [13]. Gold has been reported to enhance radiation dose in megavoltage x-ray therapy [14]–[18].

Detailed studies of gadolinium radiation dose enhancement in megavoltage radiotherapy have not been published yet. Questions like “how much dose enhancement can gadolinium-containing materials reach at the concentration level used for MRI? Or what concentration is needed to get a meaningful dose enhancement?” still need to be answered. For signal enhancement purposes in MRI, the Gd concentration does not need to be high, since signal enhancement saturation is reached when the concentration is>5 mM, or>0.8 mg/mL [19]. This study calculates the gadolinium dose enhancement in MV external beam therapy, especially for flattening filter free (FFF) beams, using a Monte Carlo method, and answers the above questions. In conventional accelerators, a flattening filter is in the beam pathway to make more attenuation in the central part of the beam so that the beam is changed from central peaked profile to a flat one. This is necessary in the three-dimension (3D) treatment technology to deliver uniform dose to the target volume. The flattening filter not only changes the beams to flat, but also hardens the beams: removes low energy photons more than high energy ones, thus makes the mean energy of the attenuated beams higher. As intensity modulated radiotherapy (IMRT) applied in clinical practice, the beam does not need to be flat and the flattening filter is thus removed from the accelerator. Accelerators with FFF beams are commercially available now. The FFF beams have a higher prevalence of lower-energy photons, since unlike with conventional beams, they are not preferentially removed by the flattening filter [20], [21]. As a result, due to the photoelectric effect dose enhancement in the presence of high-Z materials should be stronger compared to the conventional flattened beams.

The same concentration levels of gold-containing materials are also analyzed and compared. The same calculations are performed for brachytherapy using Ir-192 source. Monte Carlo methods use accurate cross section data to simulate random interactions of particles in materials. Dose calculation in radiotherapy using this method has been compared with measurements with excellent agreement, and thus is considered most accurate dose calculation algorithm in radiotherapy [22]. The other advantage of Monte Carlo method is its convenience. The calculations are performed by computers, while measurements may require expensive materials and may be labor intensive. Monte Carlo method is used in this study because it is accurate and convenient [23].

## Materials and Methods

BEAMnrc [24] version V4r2.3.2, a Monte Carlo simulation package specifically designed for radiotherapy applications which has been applied in many medical physics research projects, was used to calculate dose enhancement as a function of Gd concentration. Cross section data for the Gd materials were generated using PEGS in EGSnrc [25] based on the chemical compositions. The composition of motexafin gadolinium (MGd) used for the cross section data generation was C_52_H_72_GdN_5_O_14_. Various amounts of water, H_2_O, were added to generate Gd-containing materials of various Gd concentrations for the simulations. The Gd concentrations simulated were 1, 3, 5, 10, 15 and 20 mg/mL and were chosen based on the concentration levels presented on previous studies and the achievable concentrations with current technologies. Table 1 lists the material concentrations in the various Gd-containing material simulations. Dose in phantom was calculated using DOSXYZnrc [26].

**Table 1:** Fraction by weight of each element used in Monte Carlo simulations for various Gd concentrations.

Concentration	Gd	C	H	N	O
1 mg/mL	0.0011	0.0043	0.1107	0.0005	0.8833
3 mg/mL	0.0030	0.0119	0.1100	0.0013	0.8737
5 mg/mL	0.0050	0.0200	0.1093	0.0022	0.8633
10 mg/mL	0.0100	0.0398	0.1076	0.0045	0.8381
15 mg/mL	0.0150	0.0594	0.1058	0.0067	0.8130
20 mg/mL	0.0197	0.0783	0.1041	0.0088	0.7889

The simulated materials are mixtures of motexafin gadolinium and water.

Published phase space files for the TrueBeam linear accelerator (Varian Medical Sytems, Palo Alto, CA) flattening filter free (FFF) and conventional flattened 6MV photon beams [27] were used for the megavoltage beam simulations. The energy spectrum of the Ir-192 source was used for the high dose rate (HDR) brachytherapy modality simulations [28], [29].

The field size of the megavoltage beams used in the dose enhancement calculations was 10×10 cm^2^. A 1 cm slab of various concentrations of Gd-containing materials was inserted in a water phantom at a depth of 1.8 cm. Source to surface distance was 100 cm. The dose enhancement was calculated as the dose difference between the simulations with and without Gd-containing materials inside the slab volume along the central axis. The dose grid was 5×5×2 mm^3^, 2 mm being along the beam direction. A total of 2,650 million histories were simulated for each Gd concentration to make the uncertainty smaller than 0.5% for each voxel where the values were used for analysis.

For the Ir-192 source simulation, the source was approximated as a point source placed on top of the phantom. The top 4 mm layer of the phantom was simulated with Gd-containing materials of various concentrations and the rest was water. The dose enhancement was calculated as the dose difference inside the top 4 mm along the central axis between the simulations with Gd-containing materials and the one with water only. The dose grid size was 3×3×1 mm^3^ with 1 mm along the central axis. A total of 620 million histories were simulated in each Gd concentration.

For comparison purposes, gold (Au) was also simulated with the same weight concentrations and same source and phantom setups. The Au-containing materials were simply simulated as water with various concentrations of gold.

## Results

**Figure 1:**
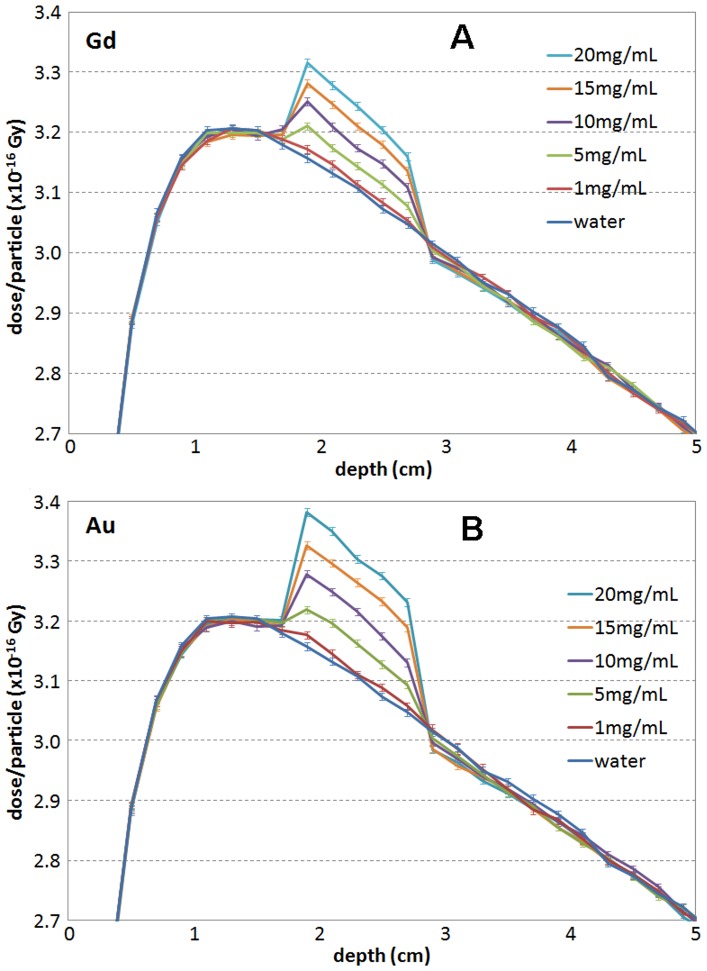
Depth dose curves simulated for a 6 MV FFF beam with (A) Gd and (B) Au slabs of various concentrations and water only. In the figure, the legends are listed in the order of dose in the slab: from 20 mg/mL, the highest, to water, the lowest.

**Figure 2:**
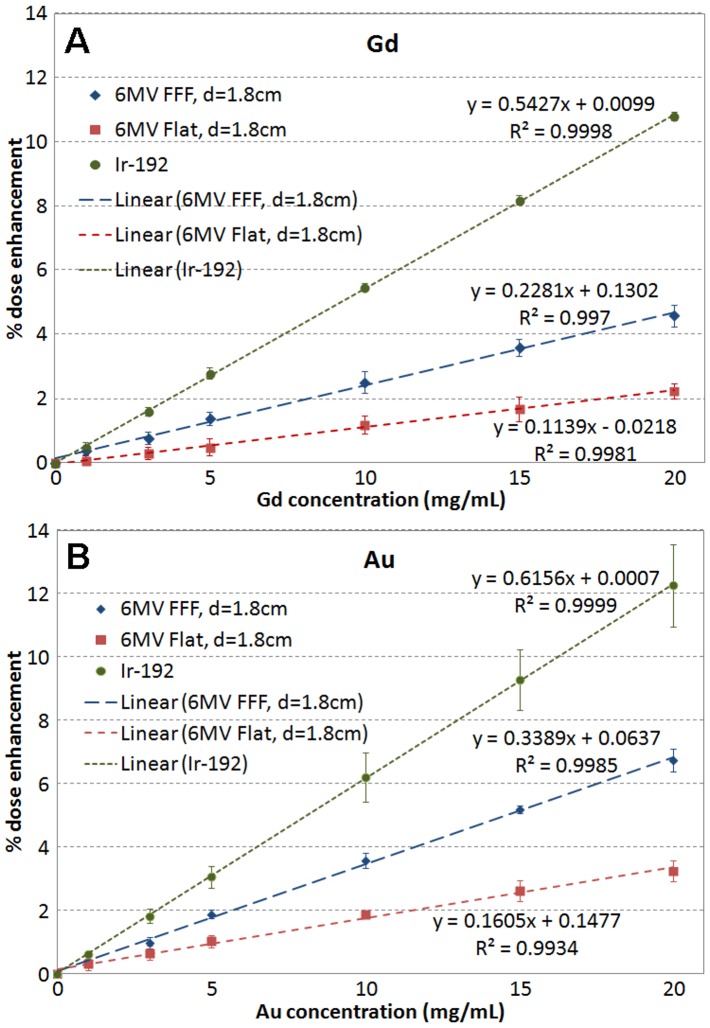
Dose enhancement versus concentration for (A) Gd and (B) Au for 6 MV FFF and flattened beams and Ir-192 source.

Figure 1 shows the simulated depth dose curves for the 6 MV FFF beam with Gd (A) and Au (B) slabs of various concentrations, as well pure water. Similar simulations were also performed for the 6 MV flattened beam. In each of the varying concentration slabs, 5 readings of dose along the central axis were obtained (Figure 1). The average differences compared to the corresponding 5 readings in water and the standard deviations were calculated for each concentration based on the 5 points. Figure 2 shows the average dose enhancement versus concentration for Gd (A) and Au (B) for the 6 MV FFF and flattened beams for the same geometry shown in Figure 1, and for the Ir-192 source. At depth of 1.8 cm, the percent mean dose enhancement for the FFF beam was 0.38±0.12, 1.39±0.21, 2.51±0.34, 3.59±0.26 and 4.59±0.34 for Gd concentrations of 1, 5, 10, 15, and 20 mg/mL respectively. The corresponding values for the flattened beam were 0.09±0.14, 0.50±0.28, 1.19±0.29, 1.68±0.39, and 2.34±0.24. For Ir-192 with direct contact, the enhanced were 0.50±0.14, 2.79±0.17, 5.49±0.12, 8.19±0.14 and 10.80±0.13.

The dose enhancement is proportional to the concentration, and the linear regression lines are shown in Figure 2. To get 1% dose enhancement, for the 6 MV FFF beams, the gadolinium concentration needs to be about 4.5 mg/mL, for the 6 MV flattened beams, 8.6 mg/mL and for the Ir-192 source, 1.9 mg/mL. For gold, the required concentrations are 3, 6.2 and 1.6 mg/mL, respectively. At 5 mg/mL of Gd, the 6 MV FFF beam gives 1.4% dose enhancement, the flattened 6MV 0.5%, and Ir-192 2.8%, while for gold the doses increase by 1.9%, 1.0%, and 3.1%, respectively.

**Figure 3:**
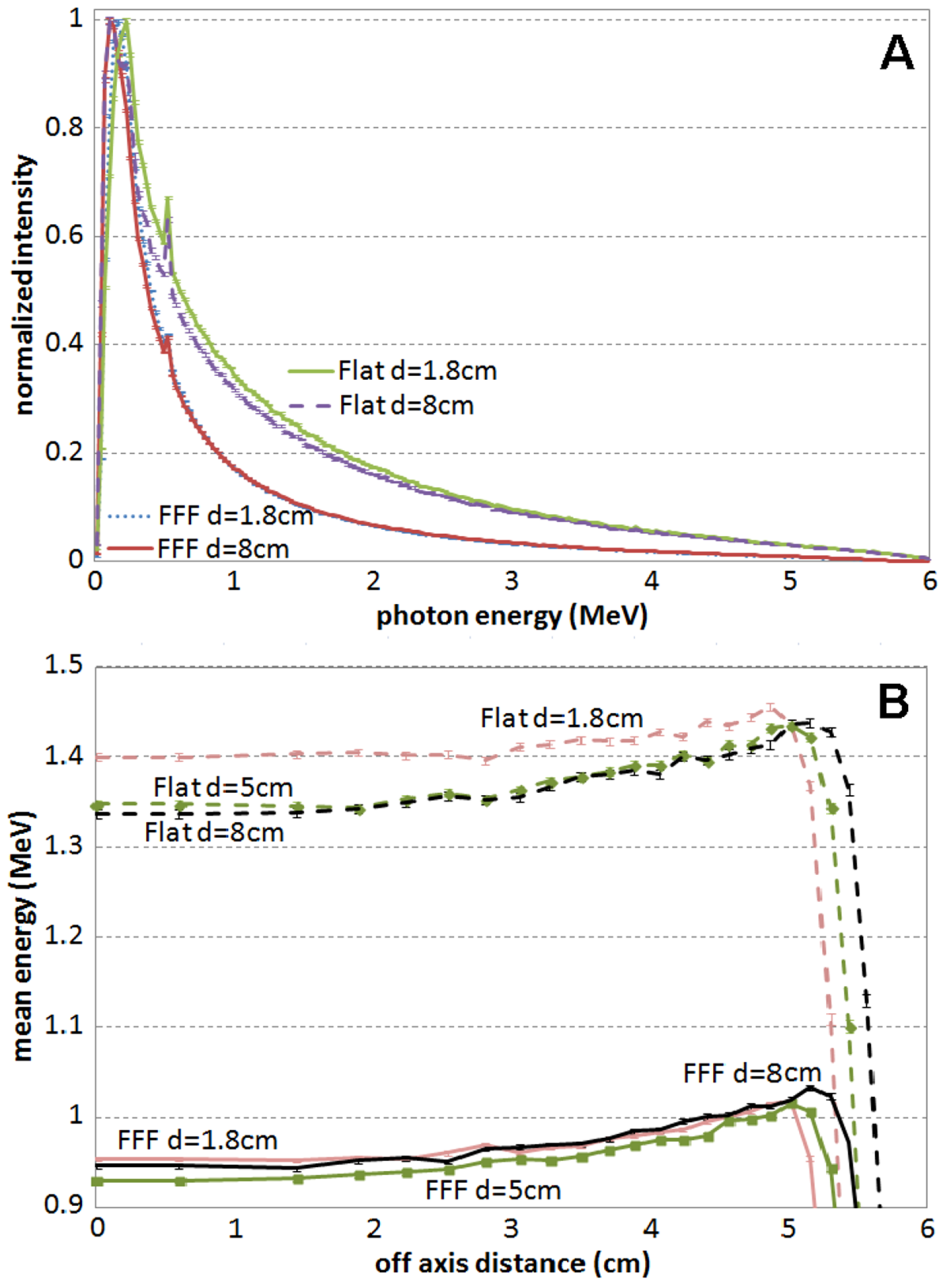
Energy spectra and mean energy versus off-axis distance for 6 MV FFF and flattened beams. (A) Energy spectra for 6 MV FFF and flattened beams at depth of 1.8 cm and depth of 8 cm and (B) mean energy versus off-axis distance for the 10×10 cm^2^ beams at the different depths for 6 MV FFF (solid lines) and flattened beams (dash lines).

**Figure 4:**
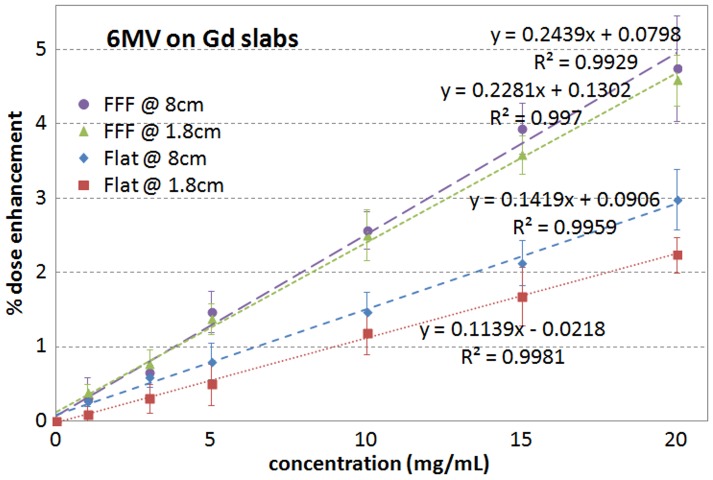
Dose enhancement comparison for 6 MV FFF and flattened beams at 1.8 cm and 8 cm depth.

Figure 3 shows the energy spectra at different depths for the 6 MV FFF and flattened beams and the mean energy versus off-axis distance for the 10×10 cm^2^ beams. The lower energy spectra in the FFF beams compared to the flattened beams is believed to be the reason that the FFF beams give higher dose enhancement than the flattened beams when high-Z materials are involved. As the energy spectrum varies little with depth for the 6 MV beams within the treatment range, the dependence of dose enhancement on the slab depth was found to be weak. Due to more difference in energy spectra at different depths for the flattened beams, the dose enhancement was found slightly higher at deep depths for the flattened beams compared to the FFF beams. Figure 4 shows the dose enhancement comparison as a function of depth for the 6 MV FFF and flattened beams.

The dose enhancement dependence on depth is more significant for the Ir-192 source (depth dependence results not presented for Ir-192 source). However, since in brachytherapy the source is inserted directly into the target volume, the depth dependence for Ir-192 would not affect clinical applications and does not need to be further discussed here.

## Discussion

The dose difference between high-Z and tissue equivalent materials is mostly caused by the difference of the photoelectric effect which dominates in the kV energy level. For Gd, the K-shell electron binding energy is 50.24 keV; for Au, it is 80.73 keV. This is why the dose enhancement is higher in microbeam radiotherapy [12] and brachytherapy [13] where the spectra are shifted towards much lower energies compared to the standard megavoltage external beam therapy, and thus more photons have energies closer to the K-shell binding energy of the high-Z materials. This study demonstrates that the dose enhancement is higher for the FFF beams compared to the conventional flattened beams, but lower than the Ir-192 brachytherapy for the same reason. It also demonstrates that gold-containing materials provide higher enhancement than gadolinium does because of the binding energy difference.

In the simulations, the chemical composition of MGd was used for the Gd-containing materials. In reality, different materials may be used. A simple mixture of water and Gd was also simulated for a few different Gd concentrations. The dose enhancement was found to be very close to the simulations using MGd. The difference of chemical composition between the two was only the small amounts of carbon and nitrogen in MGd (Table 1), which does not cause much dose deposition difference. Unless there is another high Z material involved, the dose difference between different clinically used Gd-containing materials should be minimal. This assumption also applies to the Au-containing materials.

The comparison between the same weight concentration in mg/mL of Gd and Au-containing materials slightly underestimated the Au-containing material's dose enhancement. The absorption of the tumor targeted materials in a tumor usually is measured by molar concentration in mol/L or millimol/L (mM.). To produce the molar concentration of Au equivalent to Gd, the weight concentration of Au should be 1.25 times higher. For example, if the gold concentration achieved in mice tumors is 7 mg/mL [11], then the corresponding Gd concentration should be 5.6 mg/mL. At this Gd concentration level, the dose enhancement would be 1.3%, 0.6% and 3.0% with the 6 MV FFF, flattened beams and Ir-192, respectively (Figure 2A). At the corresponding gold molar concentration level, for the same radiation sources the enhancement is 2.4%, 1.1% and 4.3% respectively (Figure 2B). Thus, purely from dose enhancement considerations, gold is a better option than Gd. However, considering the big price difference between the two metals (gold price is about 350 times higher than Gd based on the current market), Gd is a much better option economically.

The Gd concentration used in MRI or as a radiosensitizer is usually less than 1 mg/mL. The reason for the lower concentration is not because a higher concentration is not achievable, but because it is not necessary. For concentrations of higher than 5 mM, which is equivalent to 0.8 mg/mL of Gd, in MRI, the signal enhancement is saturated [19]. As a radiosensitizer, MGd concentration in the targeted tumor can be even lower, with an effective concentration around 16 µM (equivalent to 2.5 µg/mL of Gd) in cell experiments [5]. The measured MGd concentrations in clinical trials were plasma concentrations, not real concentrations in tumors [9]. The real MGd concentration inside tumor volumes as an effective radiosensitizer is still unknown, but should be close to the concentrations in the cell experiments. The original idea for this study was to examine the possible dose enhancement in radiotherapy after Gd-containing materials were used for MRI imaging or as radiosensitizers. Our results demonstrate that even at the saturation concentration in MRI imaging, the dose enhancement in external beam radiotherapy is negligible; even HDR brachytherapy using the Ir-192 source has a dose enhancement of less than 1%. However, as stated in other studies, the concentration higher than 5 mg/mL of high-Z materials, such as Gd or Au, can be achieved in the target volume with current technologies [11], [12], [14]. When Gd-containing materials are used for MRI or as radiosensitizers, higher concentrations may not provide further benefit towards the original purpose, but it could provide dose enhancement in the subsequent radiotherapy treatment. Many studies in dose enhancement of gold nanoparticles were just for this treatment purpose, in which the studied concentration was up to 30 mg/mL, which was claimed to be achievable for current nanoparticle technologies [14].

## Conclusions

At the gadolinium concentration level used in MRI, radiation dose enhancement is negligible for megavoltage radiotherapy, and even for brachytherapy using Ir-192 source. Due to lower energy spectra in FFF beams compared to the conventional flattened beams, 6 MV FFF beams demonstrate higher dose enhancement when gadolinium-containing materials are involved. At higher achievable concentration, say 5 mg/mL, 6 MV FFF beams and Ir-192 source can reach more than 1% dose enhancement.
